# *Area* Gene Regulates the Synthesis of β-Glucan with Antioxidant Activity in the *Aureobasidium pullulans*

**DOI:** 10.3390/foods12030660

**Published:** 2023-02-03

**Authors:** Kai Zhang, Wei Zhou, Wan Wang, Shanshan Zhao, Congyu Lin, Xin Ru, Jiaqi Guan, Hua Cong, Qian Yang

**Affiliations:** 1 School of Life Science and Technology, Harbin Institute of Technology, Harbin 150006, China; 2 National Engineering Research Center of Cereal Fermentation and Food Biomanufacturing, School of Food Science and Technology, Jiangnan University, Wuxi 214122, China; 3 State Key Laboratory of Urban Water Resources and Environment, Harbin Institute of Technology, Harbin 150090, China

**Keywords:** *Aureobasidium pullulans*, β-glucan, *Area*, nitrogen, regulation

## Abstract

The ability of the fungus to regulate metabolism on various nitrogen sources makes it survive and metabolize in different environments. The biomass and the β-glucan yield of *Aureobasidium pullulans* are closely associated with the nitrogen source. This study found the only GATA nitrogen source activation regulating factor *Area* in HIT-LCY^3^. In order to testify the *Area* function, we amplified its conserved domain to build a silencing vector and used the RNAi to obtain the *Area* silent strain, and then explored its effect on the phenotype of *A. pullulans* and the yield of β-glucan. We found that the biomass and β-glucan yield of the silent strain decreased significantly after culturing with different nitrogen sources, in particular when using sodium nitrate and glutamate as the source. However, the β-glucan yield increased significantly after overexpression of Area, reaching 5.2 g/L when glutamine was the nitrogen source. In addition, the strain morphology changed as well under different nitrogen sources. At last, we investigated the antioxidant activity in vitro of β-glucan and found that it has a significant clearance effect on OH·, DPPH·, and ABTS·, being best with ABTS. Therefore, this study believed that the Area gene has a certain regulation function on the synthesis of β-glucan with antioxidant activity.

## 1. Introduction

β-glucan, an acidic polysaccharide, is a bioactive polymer linked by β-glycosidic bonds in glucose. β-glucan is widely found in plants (e.g., barley, oats) and microorganisms (e.g., bacteria, fungus, yeast), among which yeast β-glucan is the most bioactive [1]. There are three major β-glycosidic bonds, namely β-1,3, β-1,4, and β-1,6. The connection modes of glycosidic bonds vary as β-glucan comes from different sources [2]. Although the mechanism of β-glucan acting as an antioxidant remains unknown, the previous studies suggested that antioxidant activity is associated with the heteromeric hydrogens’ activity in molecules that can act as free radical quenchers [3,4]. At the same time, the more the hydroxide radicals in β-glucan molecular chains are exposed, the better the scavenging effect of free radicals [5]. Recently, given its excellent bioactivity, β-glucan has been widely used in many areas, such as medicine, food, cosmetics, and feed supplement [6,7,8,9].

*Aureobasidium pullulans* (*A. pullulans*) is a polymorphic fungus with five cell morphologies (yeast, blastospore, spore of swollen, hypha, and chlamydospore), depending on the environment and growth stages [10]. *A. pullulans* can produce natural macromolecules such as pullulan and polymalic acid, and it is called black yeast due to producing melanin. Moreover, it can also produce β-glucan insoluble in water, which is comprised of β-1,3 and β-1,6 glycosidic bonds [11,12]. This β-glucan has been used as a health-promoting food additive in many countries [13,14].

Currently, the studies of β-glucan production from *A. pullulans* mainly focus on strain breeding, fermentation condition optimization, and fermenter design. Wang et al. increased the β-glucan yield by 141.7% after adding zinc sulfate (ZnSO_4_) to the culture medium [15]. An M2 strain that underwent UV mutagenesis by Naoyuk et al. can produce 9.2 g/L β-glucan [16]. The substantial cost is still the limitation of the high yield of β-glucan. Therefore, researchers focused on agricultural and industrial waste, trying to convert it into a low-cost carbon source and nitrogen source. However, there is a preference when fungi use various nitrogen nutrient substances in the environment, such as ammonia and glutamine (Gln), which are the two favorable nitrogen sources in the environment. At the same time, it inhibits other enzymes related to the metabolism of nitrogen sources and the expression of genes such as the transport protein. The phenomenon is called nitrogen metabolite repression (NMR) [17].

The fungus can make use of extensive nitrogen sources by regulating its metabolism. A comprehensive regulation system is required to ensure the effective use of nitrogen. The system controls the absorption of nitrogen sources and the gene expression needed for metabolism, assimilating into the nitrogen-containing compounds of cells through a synthetic process. The transcriptional regulation of many fungus metabolic pathways is achieved by the joint action of global transcriptional regulators and pathway-specific transcription factors [18]. The nitrogen metabolic pathway usually has a conserved GATA global regulator, which activates the regulatory pathway in the absence of a favorable nitrogen source [19,20]. When a preferred nitrogen source is missing or low in concentration (nitrogen starvation), GATA transcriptional factor activates the expression of downstream genes by combining with the gene promoter associated with non-preferred nitrogen metabolism, thereby relieving nitrogen metabolite repression and optimizing the utilization of related nitrogen sources [21]. Chi et al. found a GATA transcriptional activator *Area* with a similar function in *A. pullulans*. They verified its function and effects on the synthesis of pullulan using different concentrations of ammonium sulfate (NH_4_)_2_SO_4_ [22]. However, the utilization of other nitrogen sources and their effect on the synthesis of β-glucan was lacking.

This study first performed the separation and characterization of β-glucan and investigated the effects on *A. pullulans* growth and the yields of β-glucan under various nitrogen sources. Subsequently, *Area*, the only nitrogen-activating transcription factor, was found in the genome; it underwent a bioinformatics analysis, and its silent strain was obtained through the RNAi. We investigated the regulation of the *Area* gene on *A. pullulans* under various nitrogen sources by comparing the morphology, biomass, β-glucan yield, and the expression of genes associated with β-glucan synthesis among wide strain, silent Area strain, and overexpression Area strain.

## 2. Materials and Methods

### 2.1. Experimental Stain, Plasmid and Culture Medium

The sequences of the primers used in this study are shown in Table S1. The laboratory preserved the strains used in this study, including *A. pullulans*, *E. coli DH5α* competent, plasmid pBARGPE1-Hygro-EGFP, and pSilent 1. The culture mediums in this study were LB (tryptone 20.0 g/L, yeast extract 10.0 g/L, NaCl 10.0 g/L), YPD (glucose 20.0 g/L, yeast extract 10.0 g/L, tryptone 20.0 g/L), seed mediums (sucrose 50.0 g/L, yeast extract 2.0 g/L, NaCl 1.0 g/L, K_2_HPO_4_ 6.3 g/L, MgSO_4_ 0.2 g/L, (NH_4_)_2_SO_4_ 0.6 g/L), and fermentation mediums (sucrose 100.0 g/L, yeast extract 2.0 g/L, NaCl 1.0 g/L, K_2_HPO_4_ 6.3 g/L, MgSO_4_ 0.2 g/L, (NH_4_)_2_SO_4_ 0.6 g/L).

### 2.2. Effects of Fermentation Time and ZnSO_4_ Additive Amount on β-Glucan Yield

Different concentrations of ZnSO_4_ (0.05, 0.10, 0.15, 0.20, 0.25, 0.30 g/L) were added into the fermentation medium, repeating the above method to find out the best ZnSO_4_ addition. Strains were taken from a −80 °C freezer, inoculated into seed medium, and cultured at 28 °C for 1 day. Then, they were inoculated in a 5L airlift fermenter at a 5% inoculum size. In total, 100 mL fermented liquor was collected every 12 h to determine the pullulan yield, biomass, and β-glucan yield. The method is as follows: We first soaked the fermented liquor in an 80 °C constant temperature water bath for 20 min, and collected thallus using 10 min 5000 rpm centrifugation after cooling. The supernatant was centrifuged again (13,000 rpm, 10min) to collect supernatant and β-glucan. We then added twice the volume of ethanol into the supernatant, mixed, and allowed it to stand at 4 °C for 12 h, and centrifuged (5000 rpm, 10 min) to obtain pullulan. Subsequently, we dried the collected thallus, β-glucan, and pullulan in a 60 °C drying oven until a constant weight was reached, and calculated the biomass and the yields of β-glucan and pullulan. 

### 2.3. Infrared Spectroscopic Analysis

We powdered the isolated β-glucan in a mortar, placed a proper amount of its powder on a horizontal attenuated total reflectance, then obtained the infrared spectrogram of β-glucan through PerkinElmer 100 FT-IR Spectrometer(PerkinElmer Inc., Waltham, MA, USA).

### 2.4. Effects of Different Nitrogen Sources on Biomass and β-Glucan Yield

Different nitrogen sources (NaNO_3_, (NH_4_)_2_SO_4_, Glu, Urea, Gln) were used in the fermentation mediums for culturing *A. pullulans*. The biomass and β-glucan yield were measured according to the method described in Section 2.2, and the additive amount of different nitrogen sources was calculated based on the nitrogen contained in 0.6 g/L(NH_4_)_2_SO_4_.

### 2.5. Area Acquisition and Bioinformatics Analysis

The *Area* gene was found in the genome annotation of *A. pullulans*. The full-length gene was obtained by designing primers AreaF2/AreaR2 for amplification, and was confirmed by NCBI (https://www.ncbi.nlm.nih.gov/, accessed on 1 June 2022). SMART (https://smart.embl-heidelberg.de/smart/set_mode.cgi?NORMAL=1, accessed on 15 July 2022) was used to predict Area’s conserved domain and nuclear localization signal.

### 2.6. Construction of Area Overexpression Vector and Silencing Vector

Designed primers PSTF/PSTR and PSTF1/PSTR1 were used to amplify Area-L and Area-R fragments in Area core structural domain size 417 bp, respectively, and the pSilent 1 plasmid was digested by *XhoⅠ* and *HindⅢ* (37 °C, 3 h), followed by ligation with the fragment Area-L to obtain the intermediate vector pSt-Area-L. Next, the intermediate vector underwent double digestion by *SphⅠ* and *StuⅠ* (37 °C, 3 h) and ligated with the fragment Area-R to obtain the silent vector pSt-Area-R. The primers EGFPR1/EGFPF1 with vector homologous sequences were used for amplification of the *Area* gene, and plasmid pBARGPE1-Hygro-EGFP was digested by *EcoRⅠ* and *XhoⅠ* (37 °C, 3 h); then, we used ClonExpress MultiS One Step Cloning Kit C113 to construct the overexpression vector and named it pB-EGFP-Area. At last, double digestion and diagnostic PCR were used to verify the construction of the silencing vector pSt-Area-R and the overexpression vector pB-EGFP-Area.

### 2.7. Transformation and Verification of Mutant Strain

The PEG-mediated protoplast transformation method was employed for the above overexpression vector pB-EGFP-Area and silencing vector pSt-Area-R to transform into *A. pullulans* protoplasts, respectively. Transformants were screened on YPD plates containing 150 μg/mL hygromycin. Transformants’ genomes were extracted and sequenced to verify transformants using primer amplified ITS sequence (ITS1/ITS4) and hygromycin resistance gene (hygR/hygF).

### 2.8. Effects of Area on Biomass and β-Glucan Yield

The mutant strains obtained above were cultured in fermentation mediums containing different nitrogen sources for 6 days, and the biomass and product yields were measured following the method in Section 2.2.

### 2.9. Morphological Observation 

After 6 days of culturing in different nitrogen source mediums, 10 μL fermentation liquor was collected from the wild-type and mutant strains, respectively. These liquors were observed and photographed under a light microscope. The wild-type and mutant thallus were collected by centrifugation and fixed with 50% glutaraldehyde for 30 min after 3–5 times washing with distilled water, followed by dehydration with 50%, 75%, 80%, 90%, 95%, and 100% ethanol, respectively. All samples were treated with acetone afterward, then freeze-dried and placed on conductive tapes for observation under a scanning electron microscope.

### 2.10. Transcription Analysis of Genes Associated with β-Glucan Synthesis 

After 6 days of culturing for the wild-type and mutant strains in different nitrogen sources, the total RNA of strains was extracted using the plant total RNA kit (Tiangen Biotech, Beijing, China), and cDNA was synthesized using a reverse transcription kit (TransGen Biotech, Beijing, China). Finally, the relative expression levels of UGT and GLS were quantitatively determined using β-actin as an internal reference by QPCR kit from Promega; the kit used SYBR green as the fluorescence label, and the expression levels were assessed using 2^-ΔΔCT^.

### 2.11. Exploration Antioxidant Activity In Vitro of β-Glucan 

We weighed exactly 40 mg of β-glucan to make mother liquor at a concentration of 2 mg/mL, then diluted it successively to make β-glucan solutions for testing at concentrations of 1 mg/mL, 0.5 mg/mL, 0.25 mg/mL, 0.125 mg/mL, and 0.0625 mg/mL. This was followed by preparing vitamin C solution with the same concentrations as the positive control. It aimed to evaluate the antioxidant activity in vitro of β-glucan through the clearance tests of DPPH·, ABTS·, and OH·.

#### 2.11.1. DPPH· Clearance

We prepared 0.2 mmol/L DPPH· ethanol solution, and mixed thoroughly with different β-glucan solutions and vitamin C solution, 100 μL each. It was allowed to stand and react for 30 min away from the light. The absorbance was tested using a microplate reader at a wavelength of 512 nm.

Clearance rate of DPPH· (%) = [1 − (A1 − A2)/A0] × 100%.(1)

A0: 100 μL absolute ethanol + 100 μL DPPH· ethanol solution;
A1: 100 μL solution to be tested + 100 μL DPPH· ethanol solution;
A2: 100 μL solution to be tested + 100 μL absolute ethanol.

#### 2.11.2. ABTS· Clearance

We first prepared 7.4 mmol/L ABTS· stock solution and 2.6 mmol/L k_2_S_2_O_8_ solutions, thoroughly mixed the two solutions in a ratio of 1:1, and allowed it to stand for 12 h overnight; at last, we diluted it with distilled water until its absorbance reached 0.700 ± 0.020 at a wavelength of 734 nm, obtaining ABTS· working solution. We pipetted 100 μL of different β-glucan solutions and vitamin C solution into a 96 well-plate, immediately adding 100 μL ABTS· working solution successively, allowing it to react for 10 min fully. Then, we measured the absorbance at a wavelength of 734 nm.

Clearance rate of ABTS· (%) = [1 − (A1 − A2)/A0] × 100%.(2)

A0: 100 μL distilled water + 100 μL ABTS· working solution;
A1: 100 μL solution to be tested + μL ABTS· working solution;
A2: 100 μL solution to be tested + 100 μL distilled water.

#### 2.11.3. OH· Clearance

We pipetted 1 mL of polysaccharide solution and vitamin C solution prepared above at different concentrations, adding 1 mL of 9 mmol/L FeSO_4_ and 1 mL of H_2_O_2_ successively. We mixed it well, and incubated it at 37 °C for 10 min, adding 1 mL of 9 mmol/L salicylic acid and allowed it to react at 37 °C for 30 min, measuring the absorbance at a wavelength of 536 nm.

Clearance rate of OH· (%) = [1 − (A1 − A2)/A0] × 100%.(3)

A0: 1 mL distilled water + 1 mL FeSO4+1 mL H2O2 + 1 mL salicylic acid;
A1: 1 mL solution to be tested + 1 mL FeSO4+1 mL H2O2 + 1 mL salicylic acid;
A2: 1 mL distilled water + 1 mL FeSO4+1 mL distilled water + 1 mL salicylic acid.

### 2.12. Statistical Analysis

All data were statistically analyzed using SPSS 19.0 (IBM Corporation). Gene expression analysis was evaluated using one-way analysis of variance (ANOVA). Duncan’s multiple-range test was used to compare data means. *p* value less than 0.05 was considered statistically significant.

## 3. Results

### 3.1. Effects of Fermentation Time on β-Glucan Yield

**Figure 1:**
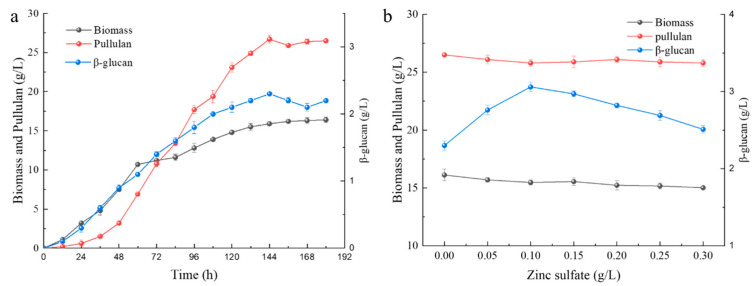
Effects of fermentation time (**a**) and ZnSO4 additive amount (**b**) on *A. pullulans* ’s biomass, pullulan yield, and β-glucan yield.

Biomass, pullulan yield, and β-glucan yield were tested during the fermentation in the 5 L airlift fermenter, with results as shown in Figure 1. The β-glucan concentration was 2.3 g/L, biomass was 24.3 g/L, and pullulan yield was 26.7 g/L at 144 h, indicating that the major production at this time is pullulan. Wang et al. found that a certain amount of ZnSO4 can increase the yield of β-glucan. In order to find the best addition amount, we performed an experiment by adding 0.05 g/L, 0.10 g/L, 0.15 g/L, 0.20 g/L, 0.25 g/L, and 0.30 g/L ZnSO4, respectively, in order to determine the right additive amount. Finally, it was found that with the increase in additional ZnSO_4_, the biomass decreased, but the yield of β-glucan increased. The β-glucan yield in this experiment reached 3.3 g/L at a ZnSO_4_ concentration of 0.1 g/L, which is similar to Wang’s result but still lower than that of strain *A. pullulans* M2 screened by Naoyuk et al. through UV mutagenesis [15,16]. 

### 3.2. FT-IR of β-Glucan

**Figure 2:**
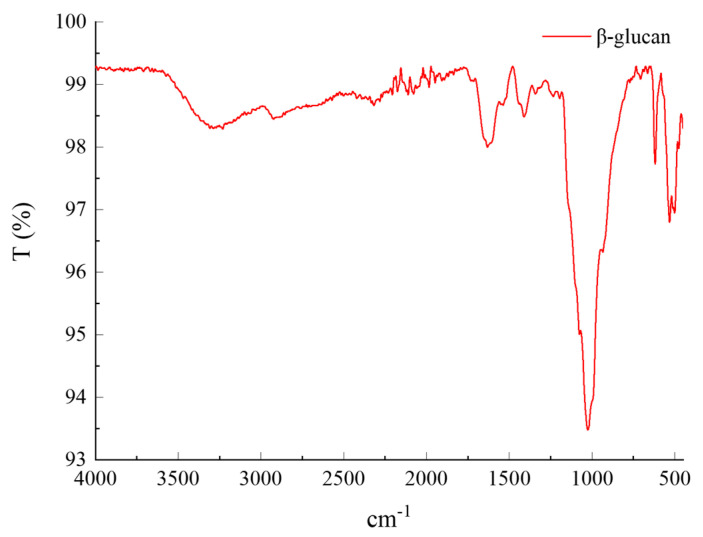
FT-IR of β-glucan.

The structure of obtained products was characterized by infrared spectroscopy, and the results are shown in Figure 2. The peaks at 3312 cm^−1^ and 1422 cm^−1^ are the stretching vibrations of carbohydrates’ intermolecular and intramolecular -OH and C-OHs. The characteristic peak of the C-H bond is at 2925 cm^−1^ [23]. Moreover, the three characteristic peaks at 1601 cm^−1^, 1161 cm^−1^, and 1015 cm^−1^ are vibrations of O-C-O, C-O-C, and C-O [24]. The most crucial characteristic peak is at 890 cm^−1^, a characteristic absorption peak of the β glucosidic bond, indicating that the isolated carbohydrate is β-glucan [25].

### 3.3. Effects of Different Nitrogen Sources on β-Glucan Yield

**Figure 3:**
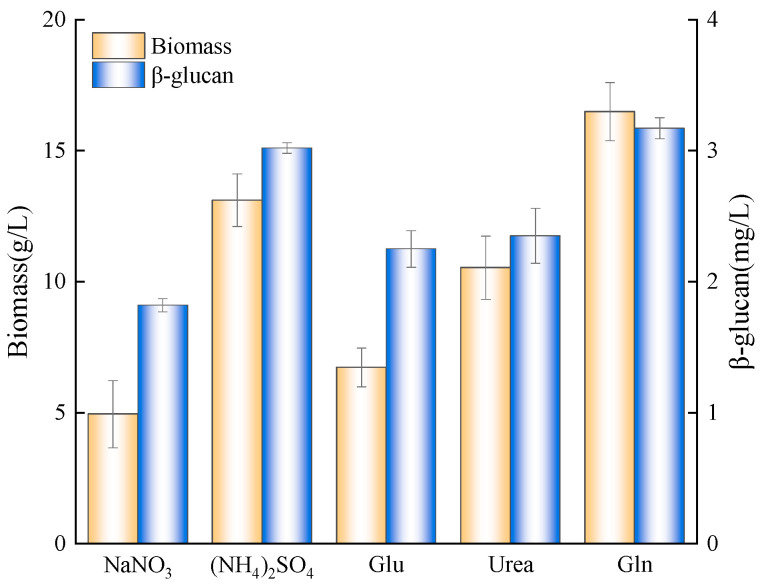
Effects of different nitrogen sources on *A. pullulans*’s biomass and β-glucan yield.

After 6 days of culturing with different nitrogen sources and adding ZnSO_4_, the biomass and β-glucan yield of *A. pullulans* are shown in Figure 3. Regarding inorganic nitrogen, the biomass and β-glucan yield of (NH_4_)_2_SO_4_ were 13.11 g/L and 3.02 g/L, both higher than that of NaNO_3_. As for organic nitrogen, the biomass under urea as a nitrogen source was higher than that of Glu but had no significant difference in β-glucan yield, lower than that of (NH_4_)_2_SO_4_. However, when Gln was a nitrogen source, the biomass was significantly higher than any other nitrogen source, and its highest β-glucan yield reached 3.17 g/L. This is due to nitrogen metabolite repression, which ensures the use of preferential nitrogen sources, increasing nutrient utilization. The phenomenon wildly exists in fungi and *saccharomyces cerevisiae* [26,27].

### 3.4. Silencing Schematics and Validation

**Figure 4:**
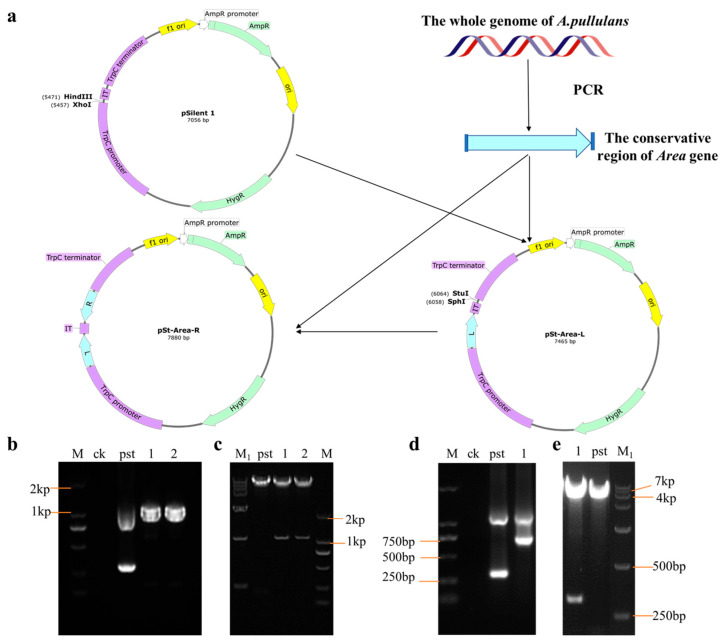
Construction and validation of Area silencing vector. (**a**) Construction strategy of Area silencing vector; (**b**) PCR validation of pSt-Area-R vector, ck is water, pst is empty vector, 1 and 2 are pSt-Area-R; (**c**) validation of double digestion (*StuⅠ* and *XhoI*) on pSt-Area-R vector, pst is pSilent1 vector, 1 and 2 are pSt-Area-R; (**d**) PCR validation of pSt-Area-L vector (primer PSR/PSF), ck is water, pst is pSilent1 vector, 1 is pSt-Area-L; (**e**) validation of double digestion (*HindⅢ* and *XhoⅠ*) on pSt-Area-L vector, pst is empty vector, 1 is pSt-Area-L.

In order to identify the function of the Area gene, we first used bioinformatics to analyze it (Figure S1) and then constructed the Area silencing vector using pSilent1 as the backbone. The process is shown in Figure 4. First, the pSilent1 vector was double digested with XhoI and HindIII, obtaining intermediate vector pst-Area-L through the ligation with the *Area* conserved region fragment from *A. pullulans* genome amplification after purification. PCR and digestion then validated the pst-Area-L vector. As shown in Figure 4e, the intermediate vector pst-Area-L (lane 1) can be digested by *XhoI* and *HindIII* into two bands: 423 bp and 7042 bp, while the pSilent1 vector has only one band of 7042 bp. Additionally, we found that pst-Area-L could amplify a band 417 bp larger than the empty vector (Figure 4d) through PCR validation, demonstrating a successful construction of the pst-Area-L vector. Subsequently, the pst-Area-L vector was digested by *StuI* and *SphI*, obtaining silencing vector pst-Area-R through reverse ligation from the *Area* conserved region fragment after purification. PCR validation is shown in Figure 4b. The successfully constructed vectors 1 and 2 can amplify a band 834 bp larger than the pSilent1 vector, which is consistent with the digestion results of pst-Area-R (Figure 4c), indicating that the Area silencing vector pst-Area-R was successfully constructed.

### 3.5. Overexpression Schematics and Validation

**Figure 5:**
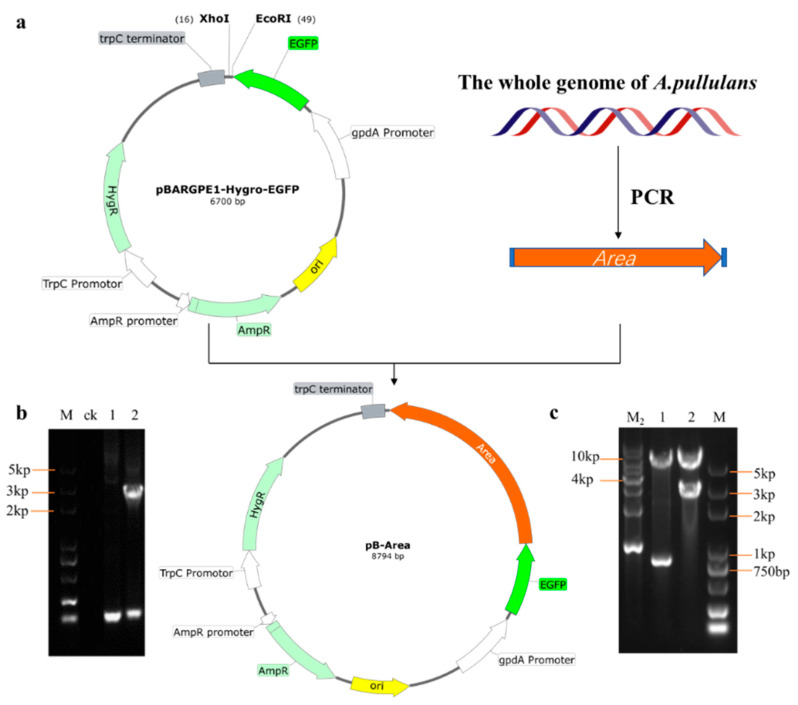
Construction and validation of overexpression vector. (**a**) Construction strategy of Area overexpression vector; (**b**) PCR validation pf pB-Area vector, ck is control, 1 is empty vector, 2 is pB-Area; (**c**) validation of double digestion (BamHⅠ and XhoⅠ) on pB-Area vector, 1 is empty vector, 2 is pB-Area.

pBARGPE-hyg-EGFP contains strong promoter gpdA, so it was selected as the backbone of the overexpression vector in this study. The pB-Area overexpression vector was obtained by ligating with Area complete genes amplified from the genome after digestion with *EcoRI* and *XhoI* and recovery. As shown in Figure 5c, it was found that lane 2 could be digested into a 2853 bp band, which is 2121 bp more than the empty vector in lane 1, similar to the full length of the Area gene. PCR validation further found that a band of 2 kb could be amplified from lane 2. It was proven to be the Area gene after recovering and sequencing, indicating a successful construction of the overexpression vector.

### 3.6. β-Glucan Production in Area Mutant Strain

**Figure 6:**
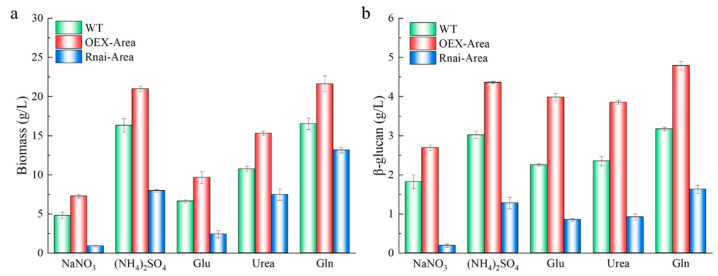
Effects of various nitrogen sources of Area mutant strains. (**a**) biomass. (**b**) β-glucan yield.

The regulation of nitrogen metabolite repression is an important mode to guarantee the efficient use of nitrogen sources by fungi. After 6 days of culturing, we found that the biomass of the overexpressed *Area* strain improved significantly under inorganic and organic nitrogen sources. With the favorable source Gln, the biomass reached its highest value of 21.6 g/L (Figure 6). However, the biomass decreased significantly after silencing the Area gene, particularly when NaNO_3_ and Glu were the nitrogen sources. The reason is that as the activator of nitrogen repression, Area can activate related genes downstream, relieving nitrogen metabolite repression [21]. In terms of β-glucan, it showed the same trend as biomass, suggesting that as a global regulatory gene, the Area gene can also regulate the synthesis of β-glucan. Further studies are required to determine whether the control is direct or indirect.

### 3.7. Expression Quantity of Genes Associated with β-Glucan Synthesis

**Figure 7:**
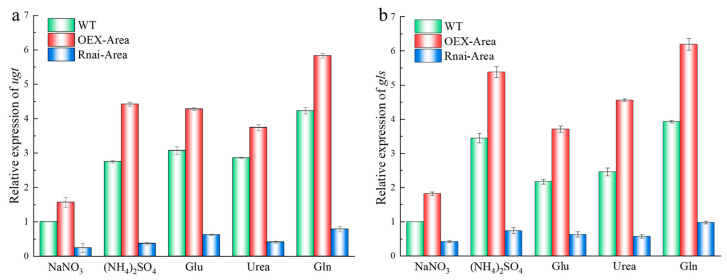
Changes of expression quantity Area of mutant strains under different nitrogen sources; (**a**) UDPG. (**b**) GLS.

As a glycosyl transferase, UGT can prolong the β-glucan chain, and β-1,3 glucan synthase (GLS) plays a key role in the synthesis of β-glucan, cell wall assembly, and fungi growth [28]. After culturing with various nitrogen sources, *A. pullulans* and its mutant strains, the expression quantity of UGT and GLS are shown in Figure 7. The expression of UGT is significantly different when comparing Gln with NaNO_3_ as the nitrogen source of WT (Figure 7a). In terms of GLS expression, it was higher when (NH_4_)_2_SO_4_ and Gln were used as nitrogen sources (Figure 7b). However, the expression of UGT and GLS significantly decreased after *Area* silencing. In contrast, the expression increased again after overexpression of *Area*, the same as the previous finding of β-glucan yield changes.

### 3.8. Micromorphology of Mutant Strains under Different Nitrogen Sources

**Figure 8:**
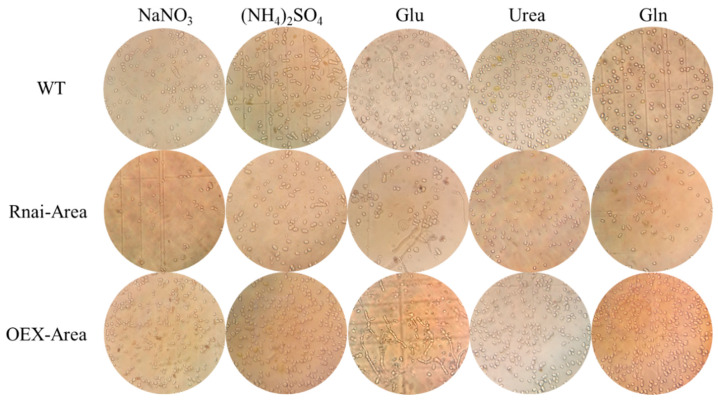
Micromorphology of mutant strains culturing with different nitrogen sources.

**Figure 9:**
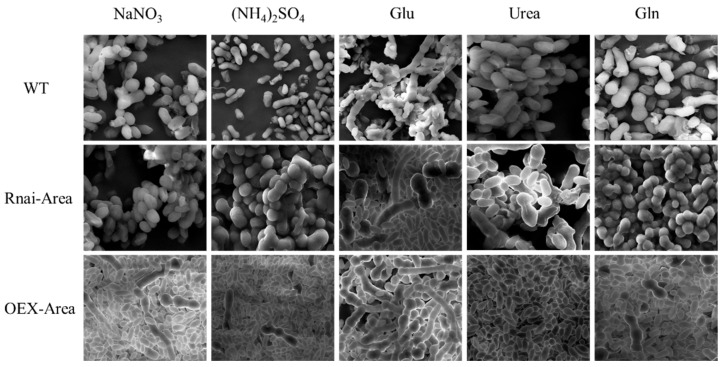
SEM of mutant strains culturing with different nitrogen sources.

In the presence of organic nitrogen Urea, the optical microscopy observed that most cellular morphologies were typically yeast-like in both WT and mutant strains. A few were swollen cells and chlamydospores, with no hypha existing (Figure 8). However, after the scanning electron microscope discovered the silencing Area, the number of chlamydospores and yeast-like cells was comparable. In contrast, the yeast-like cells became the major morphology after Area was overexpressed (Figure 9). After culturing with the preferred organic nitrogen Gln, the major morphologies of the WT strain observed in microscopy were swollen cells and chlamydospores, whereas silenced and overexpressed transformants showed the dominance of chlamydospores and swollen cells, respectively (Figure 8 and Figure 9). After culturing with Glu, the cell morphology of WT and silencing strain was increasingly diversified: yeast-like, swollen cells, chlamydospores, and hyphae. However, the number of hyphae outweighed other morphologies when Area was overexpressed (Figure 8 and Figure 9). After culturing with inorganic NaNO_3_, the primary morphologies of the WT strain were chlamydospores and swollen cells, whereas swollen and yeast-like cells were dominant after silencing and overexpression of Area. In addition, hyphae appeared in the overexpressed strain (Figure 8 and Figure 9). Last, under the culturing of preferred inorganic nitrogen (NH_4_)_2_SO_4_, the dominant morphology in the WT strain was chlamydospores. Swollen cells and chlamydospores dominated the silencing strain, and yeast-like cells dominated the overexpressed strain (Figure 8 and Figure 9). Furthermore, considering the morphology of the mutant strains at these nitrogen sources and β-glucan yield, we realized that yeast-like and swollen cells are responsible for producing β-glucan. Therefore, as the key transcriptional activator, GATA transcription factor *Area* can not only regulate the absorption and utilization of nitrogen sources but can also significantly regulate the cell morphology of *A. pullulans.*

### 3.9. Antioxidant Activity In Vitro of β-Glucan

**Figure 10:**
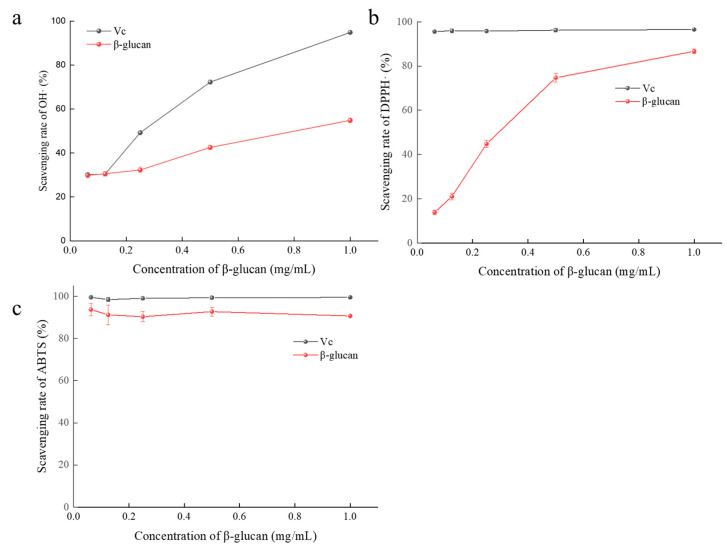
Antioxidant activity of β-glucan. (**a**) The clearance rate of β-glucan on OH·; (**b**) the clearance rate of β-glucan on DPPH·; (**c**) the clearance rate of β-glucan on ABTS·.

The study found that the β-glucan from microorganisms has a certain antioxidant activity [1,29,30]. We selected Vc as the control to test the antioxidant activity of β-glucan produced by A. pullulans and evaluated its clearance on OH·, DPPH·, and ABTS·. The clearance effect of β-glucan on OH· was shown in Figure 10a. With the increase in concentration in Vc and β-glucan, the clearance rate of OH· increases, peaking at 54.8%. The clearance rate of β-glucan, however, is lower than Vc all the time. The clearance rate of β-glucan on DPPH· is shown in Figure 10b. With the increase in concentration in β-glucan, the clearance rate of DPPH· increases, peaking at 86.6%, still lower than Vc. However, ABTS· shows its excellent effect (Figure 10c). It reached a clearance rate of 86.1% at the lowest concentration of 0.065mg/mL, comparable to Vc.

## 4. Discussion

In recent years, the research interest in bio-based polymers has been increasing, especially regarding microbial extracellular polysaccharides with good physical and chemical properties and biological activities. Although all fungi have cell walls, the polysaccharide structure of cell walls varies with the species of fungi. In addition, the cell wall of the same fungus can also change among morphological types in different life cycles [31]. β-glucan is mainly composed of β-1,3, β-1,4, and β-1,6 glycosidic bonds, which are essential for maintaining the integrity and morphology of the cell wall. The water-insoluble β -glucan produced by *A. pullulans* is mainly composed of β-1,3 and β-1,6, glycosidic bonds [11,12]. At present, the production of *A. pullulans* β -glucan is still under theoretical research, and the reasons that restrict its mass production are high substrate cost and low yield. In order for A. pullulans to produce β-glucan by fermentation with cheaper nitrogen sources, it is necessary to solve the NMR phenomenon in the presence of various nitrogen sources [22].

Fungi can make use of large sources of nitrogen by regulating their metabolism. To ensure the efficient use of nitrogen, a comprehensive regulatory system is needed. This system controls the expression of genes required for the absorption and metabolism of nitrogen sources, and assimilates nitrogen-containing compounds into adult cells through a synthetic process [18]. The utilization of nitrogen sources by fungi is highly regulated by transcription, and the transcription factor responsible for regulation is named a Gata-type zinc finger transcription factor due to its structure containing zinc finger and its specific recognition of target gene promoter sequence 5 ‘-HGATAR-3’ (H represents A, T or C, R represents A or G) [32]. GATA-type transcription factors, related to nitrogen source metabolism, have positive regulatory factor Area and negative regulatory factor AREB [22]. Studies have shown that Area genes can regulate the secondary metabolism of organisms; for example, Fumonisin B1, a secondary metabolite of *Fusarium verticillioides*, was biosynthetic and *Gibberella fujikuroi* guided the biosynthesis of gibberellin [20,33]. In this study, we first constructed the silent strain of nitrogen source regulator Area by RNAi technology and discussed the effects of different nitrogen sources on the growth, morphology, and β -glucan synthesis of *A. pullulans*. It was found that the growth and development of the silent Area strain and the synthesis of β-glucan were inhibited in both organic and inorganic nitrogen cultures, but the biomass and β-glucan yield of the silent Area strain were significantly increased after overexpression of Area. The reason is that overexpression of the nitrogen metabolism activator Area can activate the expression of downstream genes by binding to the promoter of related genes of non-preferred nitrogen metabolism, thereby relieving nitrogen metabolite repression and improving the utilization of non-preferred nitrogen sources [21]. *A. pullulans* has a variety of cell morphologies, and the composition of the culture medium is closely associated with the morphology of the strain. The previous study found that yeast-like and swollen cells were the primary morphologies for pullulan production, while chlamydospores and hyphal morphologies were the main contributors to melanin production [34,35]. *A. pullulans* can use multiple compounds as nitrogen sources but presented different cell morphologies under different nitrogen sources [36]. It was also found that yeast-like cells and swollen cells were the main forms of β -glucan production, and Area also had a certain regulatory effect on the cell morphology of A. pullulans cultured in different nitrogen sources. In addition, after overexpressing Area strain was cultured in GlFig as the nitrogen source, the expression level of UGT and FKS, related to β -glucan synthesis, was the highest, with the highest yield of 5.2 g/L. Finally, it was found that β-glucan had good scavenging effects on OH·, DPPH·, and ABTS, among which ABTS was the best. This study will provide a theoretical basis and guidance for A. pullulans to efficiently produce β -glucan with antioxidant activity by using different nitrogen sources.

## Data Availability

The data presented in this study are available on request from the corresponding author.

## Supplementary Materials

The following supporting information can be downloaded at https://www.mdpi.com/article/10.3390/foods12030660/s1, Figure S1: The conserved domain of Area of A. pullulans; Table S1: primers used in this study.
