# Supplementary material for: Area Gene Regulates the Synthesis of β-Glucan with Antioxidant Activity in the Aureobasidium pullulans
Source: Foods. 2023 Feb 3;12(3):660. doi: 10.3390/foods12030660 (PMC9914807; doi:10.3390/foods12030660)
Supplement: Supplementary file 1 [file foods-12-00660-s001.zip › foods-2155504-supplementary.pdf]

Figure S1

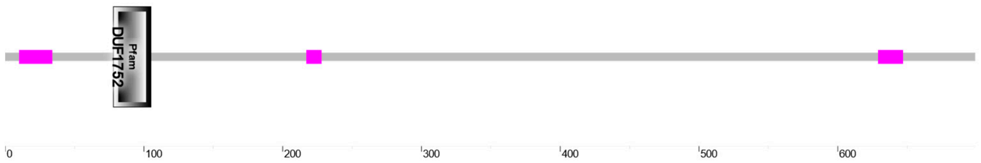

Figure S1. The conserved domain of Area of A. pullulans.

Table S1

Table S1. primers used in this study.

Primers	Sequence
AreaF2	5’-ATGACATCGCCGCGCACCTTTGC-3’
AreaR2	5’-TCAGCGGACGTACGACGCCGT-3’
EGFPF1	5’-AGCTGTACAAGTAAGAATTCATGACATCGCCGCGCACCTTTGC-3’
EGFPR1	5’-GTACCGGGCCCCCCCTCGAGTCAGCGGACGTACGACGCCGT-3’
pBEGFPF	5’-TCGACCTCGACTCTAGAGGATCC-3’
pBEGFPR	5’-TCAGCGGACGTACGACGCCGT-3’
PSTF	5’-GGCTCATTCGTATCCTCCAGCAAGCTTGACTGTCTCAGCTGTGCAA-3’
PSTR	5’-CCCAAGCATCGATACCGTCGACCTCGAGTCTTCTTCGACGACCTCTTC-3’
PSTF1	5’-GAACGGCAGATCTTCGCATGCGACTGTCTCAGCTGTGCAA-3’
PSTR1	5’-CGGGGCCCAGGTACCACAGGCCTTCTTCTTCGACGACCTCTTC-3’
PSF	5’-TTTATTTCAGATTCAATCTGACTTACCTAT-3’
PSR	5’-AGATTCGTCAAGCTGTTTGATGATTTCAGT-3’
ITS1	5’-TCCGTAGGTGAACCTGCGG-3’
ITS4	5’-TCCTCCGCTTATTGATATGC-3’
hygF	5’-CTCCATACAAGCCAACCAC-3’
hygR	5’-GAAAAAGCCTGAACTCACC-3’
